# Draft Genome Sequence of *Pasteurella multocida* Isolate P1062, Isolated from Bovine Respiratory Disease

**DOI:** 10.1128/genomeA.01254-15

**Published:** 2015-10-22

**Authors:** Juan E. Abrahante, Samuel S. Hunter, Samuel K. Maheswaran, Melissa J. Hauglund, Fred M. Tatum, Robert E. Briggs

**Affiliations:** a Department of Veterinary and Biomedical Sciences, University of Minnesota, St. Paul, Minnesota, USA; b Institute for Bioinformatics and Evolutionary Studies, University of Idaho, Moscow, Idaho, USA; c National Animal Disease Center, Agricultural Research Service, U.S. Department of Agriculture, Ames, Iowa, USA

## Abstract

Here, we report the draft genome of *Pasteurella multocida* isolate P1062 recovered from pneumonic bovine lung in the United States in 1959.

## GENOME ANNOUNCEMENT

*Pasteurella multocida*, a Gram-negative bacterium, is a pathogen which affects many species of agricultural importance including cattle, sheep, goats, swine, and poultry. In cattle it is considered an opportunistic pathogen associated with respiratory disease in beef and dairy cattle (1). The bacterium is also found as a commensal in the respiratory tract of apparently healthy cattle. In the United States, *P. multocida* serotype A:3 is the most common isolate associated with this disease (1). The P1062 strain of *P. multocida* serotype A:3 presented in this study originated from the pneumonic lung of a Holstein-Friesian calf that died with respiratory disease in 1959 (2). It is pathogenic to cattle and experimental pneumonic pasteurellosis can be reproduced by intra-pulmonic instillation with logarithmic-phase culture of the isolate. Antimicrobial resistance among bacterial bovine respiratory disease pathogens is of growing concern (3, 4), and multidrug-resistant isolates of *Pasteurella multocida* and *Mannheimia haemolytica* were recently sequenced (5, 6). The genome sequencing of this isolate was undertaken to facilitate the study of species specificity among known virulent *P. multocida* and to provide insight into the acquisition of antimicrobial resistance.

The genome sequencing of *P. multocida* was achieved using 3 platforms: Roche 454 GS FLX Titanium resulting in 30-fold coverage; Illumina GA IIx resulting in 13-fold coverage; and PacBio RS resulting in 9-fold coverage. Illumina reads were used to error-correct the PacBio reads using CLC-Genomics Workbench v6.0.2. A hybrid assembly using the CLC software was performed and the resultant contigs were aligned to an optical map (OpGen, MapSolver software, Gaithersburg, Maryland) to confirm the assembly and generate a single scaffold. Reiterative alignments of the 454 and corrected PacBio reads >500 bp against the scaffold, using the CLC software, failed to close the remaining 5 gaps in the single scaffold. The P1062 genome scaffold consists of 2.70 Mb and 5 contigs with a total length of 2.51 Mb, a G+C content of 40.3%, *N*_50_ of 140,532 bp, and 100% of contigs >500 bp.

Annotation of the genome was accomplished with the NCBI Prokaryotic Genome Annotation Pipeline revision 2.2. The genome contained a total of 2,456 genes including 2,324 predicted protein-encoding genes, 58 pseudogenes, 19 rRNA, and 53 tRNA genes. Two CRISPR arrays were detected. In contrast to the multi-resistant *P. multocida* isolate 36950 (7), an intact tRNA^leu^ is present at the site of ICEPmu1 integration. BLAST analysis revealed no significant homology in the P1062 chromosome with ICEPmu1. The determination of genes involved in host specificity of *P. multocida* will require additional analysis.

### Nucleotide sequence accession numbers.

This whole-genome shotgun project has been deposited at DDBJ/EMBL/GenBank under the accession number ASZP00000000. The version described in this paper is the second version, ASZP02000000.
